# The potential of cannabinoids in managing cancer-related anorexia in older adults: a systematic review of the literature

**DOI:** 10.1016/j.jnha.2024.100299

**Published:** 2024-06-24

**Authors:** Chiara Ceolin, Marina De Rui, Adele Ravelli, Mario Virgilio Papa, Maria Devita, Giuseppe Sergi, Alessandra Coin

**Affiliations:** a Geriatrics Division, Department of Medicine (DIMED), University of Padua, via Giustiniani 2, 35128 Padova, Italy; b Department of General Psychology (DPG), University of Padua, Via Venezia 8, 35131 Padova, Italy

**Keywords:** Aging, Older adults, Cannabinoids, Nutrition

## Abstract

**Background and objectives:**

Anorexia of aging (AoA) is a prevalent geriatric syndrome characterized by a multifactorial decline in appetite and reduced food intake associated with the aging process. This systematic review aims to investigate the use and outcomes of cannabinoids in addressing AoA, with the goal of providing a comprehensive understanding and discussing their potential integration into daily clinical practice.

**Methods:**

A thorough search of databases (Embase Ovid, Scopus, PubMed, Cochrane Library, and Web of Science) identified 6100 studies. After eliminating duplicates and screening titles and abstracts, 25 studies underwent full appraisal. Two reviewers assessed inclusion suitability, and study methodologies were evaluated using the Newcastle-Ottawa Scale (NOS) for observational studies and the modified Jadad Scoring Scale for randomized controlled trials. Ultimately, six studies published between 2002 and 2019, involving 869 participants, were included in the review.

**Results:**

Out of the 6 fin. l papers selected, 5 were randomized trials, and 1 was a prospective study. Megestrol acetate (800 mg/d) proved to be more effective than dronabinol 2.5 mg twice a day in increasing appetite. Nabilone (at a dosage of 0.5 mg per day) did not show superiority over placebo in alleviating symptoms such as pain, nausea, loss of appetite, and weight. However, with a double dosage followed by 1.0 mg/6 weeks, after eight weeks of treatment, patients recorded a significant increase in calorie intake and carbohydrate consumption compared to the placebo group, with some patients also experiencing substantial weight gain. Regarding delta-9-tetrahydrocannabinol (THC), a weight increase of ≥10% was observed in 17.6% of patients with doses of 5 mg or 10 mg capsules daily, without significant side effects. Additionally, patients treated with THC 2.5 mg reported improved chemosensory perception and increased appetite before meals compared to placebo. No significant side effects were reported in older adults taking cannabinoids.

**Conclusions:**

Cannabinoids offer promise in enhancing the quality of life for older individuals with active neoplastic disease. However, to establish comprehensive guidelines, further research with larger sample sizes is essential. Only through this approach can we fully grasp the potential and application of cannabinoids in addressing the nutritional concerns associated with neoplastic diseases.

## Highlights

• Anorexia of Aging (AoA) is a highly prevalent syndrome among older adults.
• AoA links to adverse outcomes, such as alterations in functional autonomy, mood, cognition.
• In patients with active neoplasia, cannabinoids increase weight, restore hunger and appetite.
• Limited studies in the general older population restricts results applicability.

## 1 Introduction

Anorexia of aging (AoA) is a common geriatric syndrome describing the multifactorial loss of appetite and decreased food intake that can be attributed to the aging process [1]. Because of its high prevalence (up to 63% depending on the setting and assessment methods) and its burden, it is recognized as an urgent and so far unmet clinical need among older adults [2].In fact, if not appropriately recognized and treated, AoA can lead to malnutrition [3] and increased mortality [4]. Correlates of AoA include body wasting (i.e., sarcopenia and cachexia) and decreased functional independence, with increased risk of falls and impaired mobility [5,6]. AoA and weight loss are also associated with depression, cognitive impairment, and reduced sleep efficiency [7].

The link between AoA/weight loss and clinical adverse events is complex and bidirectional: on the one side, reduced food intake and weight loss can contribute to etiology of morbidities, bringing to malnutrition, frailty and worse functional status [8]. On the other side, severe and chronic diseases common in late life may induce appetite and weight loss. This is the case of gastrointestinal diseases, infections, heart failure, inflammatory diseases, and cancer [9]. Finally, decreased appetite and weight loss may occur as side effects of polypharmacy [10].Treatment options include, first of all, the management of all reversible contributing factors, through the revise of pharmacologic therapies, food manipulation (e.g., flavor enhancement, modification of palatability and presentation) and environmental strategies as promoting conviviality and an adequate time dedicated to meals [1]. The effectiveness of pharmacological interventions, conversely, is controversial. None of the drugs tested for treating AoA to date has achieved results so encouraging as to be recommended in everyday clinical practice, primarily because of the significant side effects associated with their long-term use (as in the case of anabolic steroids, metoclopramide, megesterol, meclobemide) or the lack of physical and functional improvement associated with weight gain, as observed with corticosteroids and growth hormone [1,11]. Among the pharmacological possibilities under evaluation, potential has been demonstrated by cannabinoids, a class of drugs derived from the active component of the *Cannabis Sativa* plant, delta-tetrahydrocannabinol (THC). The hyperphagic effects of THC are known anecdotally and have been reported in studies involving healthy participants [12]. Cannabinoids have also shown promising results in stimulating appetite in AIDS patients [13] and in controlling chemotherapy-induced nauseaand vomiting in cancer patients [14]. Several formulations of cannabis with different pharmacokinetics and pharmacodynamics are available, which may demonstrate varying efficacy in the multifactorial etiology of appetite loss.

However, current conclusions regarding the use of cannabinoids for appetite stimulation and food intake in geriatric patients are only preliminary, due to mixed results regarding their actual effectiveness and documented side effects [15,16].

Thus, the aim of this systematic review is to investigate the use and results obtained through the application of cannabinoids for the management of AoA, in order to provide a more definite picture and discuss about the possibility of their current actual use in daily clinical practice.

## 2 Methods

### 2.1 Systematic review tool

This review adheres to PRISMA (http://www.prisma-statement.org/) and Meta-Analysis of Observational Studies in Epidemiology (MOOSE) guidelines [17].

### 2.2 Study identification

The Cochrane Library, Embase Ovid, PubMed, and Web of Science databases were searched for the terms “Cannabis”, “Dronabinol”, “Nabiximol”, “Epidolex”, “Bedrocan”, “Nabilone” or“Desanabinol” and “anorexia”or“appetite stimulants” from any date to November 2023. Only papers and reviews in English were selected. The articles of interest consisted ofstudies includingolderadults aged 60 years or over. References cited in the selected papers were examined to identify any other potential articles. After selecting the articles (carried out by first reviewer M.V.P.), the whole process was repeated and confirmed by a second reviewer (C.C.) to ensure the validity of inclusion. Differences of opinion were discussed until consensus was reached on the inclusion or exclusion of a study through a third reviewer (A.R.).

### 2.3 Risk of bias assessment

With respect to the methodology of included studies, quality assessment was conducted independently by 2 reviewers (M.V.P. and C.C.) following Newcastle-Ottawa Scale (NOS) criteria for observational studies. The quality of the included randomized controlled trials (RCTs) was evaluated independently by two review authors (M.V.P and C.C) with the modified Jadad Scoring Scale. The modified Jadad Scale is composed of four domains according to literature: randomization (0–2 points), concealment of allocation (0–2 points), double blinding (0–2 points), withdrawals, and dropouts (0–1 points). An overallscore of 4–7 is indicative of a high-quality study, and 1–3 of low-quality.

### 2.4 Selection criteria

Inclusion criteria were: (1) articles on older adults aged ≥60 years, thus including also “young old” people; and (2) articles dealing with any use of cannabinoids for stimulating appetite.

Exclusion criteria were: (1) case reports, abstracts, letters, and editorials; (2) studies not written in English; or (3) animal model studies.

### 2.5 Data extraction

The titles and abstracts of the selected articles were screened for relevance, and the following data were extracted from them: (1) study design; (2) country; (3) sample size; (4) median/mean age of participants; (5) type of intervention (including type of cannabinoid used); (6) main outcome; (7) adverse or collateral events recorded; (8) study limits.

## 3 Results

**Fig. 1:**
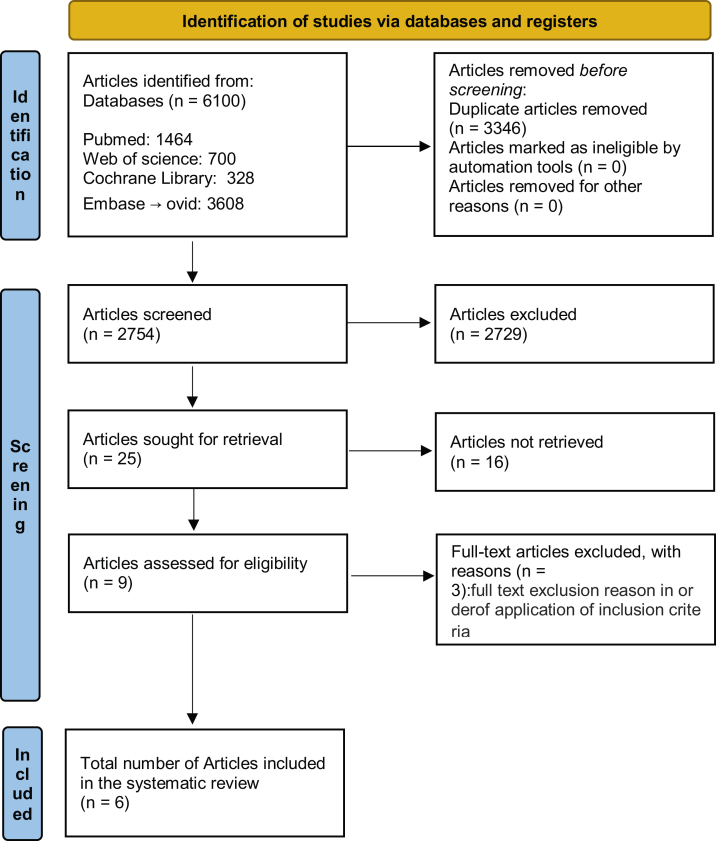
PRISMA flow diagram of the study selection process with reasons for inclusion and exclusion.

A total of 6100 studies were identified from the database searches, of which 3346 duplicates were excluded. After reviewing the titles and abstracts, 2729 studies were discarded because they did not conform to the inclusion criteria; of the remaining 25 a further 19 were discarded (inappropriate populations, missing data, etc.) leaving 6 papers, the full manuscripts of which were assessed for eligibility (Fig. 1).

The quality assessments of these 6 studies are reported in detail in Table 1, Table 2. The 5 clinical trials included [[18], [19], [20], [21], [22]] were considered to be high-quality study with an average score of 5.8. For a single observational study included [15], we performed the methodological quality analysis using the Newcastle-Ottawa Scales for observational studies, with a result of 6, indicative of a high-quality study.

**Table 1:** Study quality assessment using Newcastle-Ottawa scale for observational studies.

Author/year	Selection				Comparability (matched analysis)		Outcomes		NOS score
	Consecutive or obviously Representative series of cases	Representativeness of exposed cohort	Ascertainment of exposure	Demonstration that outcome of interest was not present at the start of study		Assessment of outcome	Follow up long enough for the outcome	Adequacy of follow-up of cohorts	
Bar-Sela G. (2019) [15]	*	*	*	*	*	*	–	–	6

*NOS* Newcastle Ottawa quality assessment scale.

*Each asterisk represents if individual criterion within the subsection was fulfilled.

**Table 2:** Quality assessment of included trials using the Jadad scale.

Corresponding author	Was the research described as randomized?	Was the approach of randomization appropriate?	Was the research described as blinding?	Was the approach of blinding appropriate?	Was there a presentation of withdrawals and dropouts?	Was there a presentation of the inclusion/exclusion criteria?	Was the approach used to assess adverse effects described?	Was the approach of statistical analysis described?	Total
Jatoi A. (2002) [18]	1	1	1	1	0	1	0	1	6
Côté M. (2016) [19]	1	1	1	1	0	1	0	1	6
Brisbois et al. (2011) [20]	1	1	1	0	1	1	0	1	6
Strasser F. (2006) [21]	1	1	1	0	1	1	1	1	7
Turcott J. (2018) [22]	1	0	1	0	0	1	0	1	4

### 3.1 Study characteristics

Of the 6 papers selected, 5 were randomized trials, and 1was a prospective study. All the studies dealt with use of cannabinoids for appetite stimulation in older adults and met the aforementioned inclusion criteria. A total of 869 participants were involved in the selected studies, which were carried out in the USA [18], Canada [19,20], Germany [21], Mexico [22] and Israel [15] and were published between 2002 and 2019.

Table 3 lists all the studies selected and their main findings. All studies focused on cancer patients.

**Table 3:** Selected studies and their main findings.

First author (Year) and reference	Type of article/study design	Country	mean/median AGE	Sample Size	Intervention (type of cannabinoid)	Outcome	Adverse or collateral event	Study LIMITS
Jatoi A. (2002) [18]	Randomized trial	USA	66 ± 10	469	(1) oral megestrol acetate 800 mg/d liquid suspension plus placebo (2) oral dronabinol 2.5 mg twice a day plus placebo, or (3) both agents. The outcome was assessed thought questionnaires were performed weekly and monthly.	Within the megestrol acetate group, 75% of patients reported that this agent increased their appetite, whereas 49% of patients the dronabinol group reported such improvement. The combination arm resulted in 66% of patients’ reporting an improvement in appetite when compared with the megestrol acetate arm.	Toxicity incidence that included monitoring for nausea, vomiting, neurocortical dysfunction, edema, ascites, pleural effusion, or thromboembolic phenomena was not statistically different between treatment groups	Higher dose of dronabinol
Côté M. (2016) [19]	Randomized trial	Canada	63.5	56	Nabilone (0.5 mg) 1 pill at bedtime for one week. For the second week, the dose was increased to 2 pills a day. From the third week, the dose was adjusted to a maximum of 4 pills a day. The corresponding procedure was done in the control group, using the placebo. The total follow-up period ranged from 9 to 11 weeks.	Nabilone was not better than placebo for relieving symptoms like pain (p = 0.6048), nausea (p = 0.7105), loss of appetite (p = 0.3295), weight (p = 0.1454).	There was no difference in the occurrence of any of the adverse effects of nabilone, including drowsiness (P = .3166), anxiety (P = .9163), and xerostomia (P = .8341).	Small sample size. Single center study.
Strasser F. (2006) [21]	Randomized trial	Germany	61 ± 12	289	Cannabis extract (CE), delta-9-tetrahydrocannabinol (THC) and placebo (PL). After 7–14 days of baseline assessment, eligible patients were randomly assigned to treatment with PL, CE, or THC for 6 weeks. Patients received a 2-week supply of capsules to take orally twice daily (1 h before lunch and before dinner or at bedtime, preferably with milk) containing CE (2.5 mg THC and 1 mg CBD) or only THC (2.5 mg) or placebo.	No differences in patients’ appetite were found either between cannabis extract, delta-9-tetrahydrocannabinol, and placebo or between cannabis extract and delta-9-tetrahydrocannabinol at the dosages investigated	Hazard rate: vomiting 0.06, fatigue 0.04.	Lack of intrapatient dose escalation of CE to test whether CBD may protect from dose-limiting THC adverse effects.
Turcott J. (2018) [22]	Randomized trial	Mexico	61	33	Nabilone (0.5 mg/2 weeks followed by 1.0 mg/6 weeks)	Nabilone is an adequate and safe therapeutic option to aid in the treatment of patients diagnosed with anorexia. After 8 weeks of treatment, patients who received Nabilone increased their caloric intake (342-kcal) and had a significantly higher intake of carbohydrates (64 g) compared to patients receiving placebo (p = 0.040).	Common Terminology Criteria for Adverse Effects grade 3 nausea was only observed in the placebo group, while the experimental group reported only grade 2 or less events at 8 weeks post-treatment.	Small sample size
Bar-Sela G. (2019) [15]	Prospective observational study	Israel	66	11	The planned treatment was 2 × 10 mg per 24 h for six months of tetrahydrocannabinol (THC) 9.5 mg and cannabidiol (CBD) 0.5 mg. If patients suffered from side effects, dosage was reduced to 5 mg × 2 per day (THC 4.75 mg, CBD 0.25 mg).	Weight increase of ≥10% in 3/17 (17.6%) patients with doses of 5mgx1 or 5mgx2 capsules daily, without significant side effects. All patients who were involved in the study for 4.5 months reported an increase in appetite, as did 83% of the patients who completed the study.	No significant side effects reported.	Most patients suffered from various types of advanced cancer and received heavy oncological treatments at the time of the study.
brisbois et al. (2011) [20]	Randomized trial	Canada	67.0 ± 10.9	21	Patients were randomized to receive oral capsule of delta-9-tetrahydrocannabinol THC (dronabinol 2.5 mg) or placebo. Patients startedon THC 2.5 mg or placebo once daily for the first 3 days. The dose was increased toTHC 2.5 mg or placebo twice daily onthe fourth day. Patients had the option to increase their drugdose to a maximum of 20 mg/day	THC-treated patientsreported improved chemosensory perception and food ‘tasted better’.Premeal appetite and proportion of calories consumed as protein increased compared with placebo. QOL scoresand total caloric intake were improved in both THC and placebo groups.	No significant difference between groups.	Small sample size

Abbreviations: QOL, Quality of Life; THC, delta-9-tetrahydrocannabinol; CBD, cannabidiol; CE, Cannabis extract.

Two studies recruited patients suffering from cancer-related anorexia-cachexia syndrome (CACS) [15,21]. Strasser et al. conducted a comparative analysis investigating the impact of cannabis extract (CE), delta-9-tetrahydrocannabinol (THC), and placebo (PL) on appetite and quality of life in cancer patients afflicted by CACS [21]. 243 patients experiencing in voluntary weight loss of at least 5% within the past 6 months without explanation by other diseases or recent surgery were recruited (mean age 61 years). Patients were randomly assigned to treatment with PL, CE (2.5 mg THC and 1 mg CBD), or THC (2.5 mg) for 6 weeks. Active treatment demonstrated a significant improvement in appetite stimulation, quality of life domains and body weight compared to the placebo. In patients with tumors other than hematologic-lymphogenic, head, neck, and lung, or gastrointestinal-urogenital, appetite scores higher than the average for the entire population were observed. Finally, no significant differences were observed in adverse events between the groups. Also, Gil Bar-Sela and colleagues conducted a study focusing on the impact of cannabinoids on the management of CACS [15]. The study involved 11 patients with advanced cancer, characterized by a median age of 66 years. These patients exhibited a weight loss of at least 5% over the preceding 2 months and a concurrent loss of appetite. The intervention consisted of a six-month treatment regimen using a combination of tetrahydrocannabinol (THC) and cannabidiol (CBD) encapsulated together. The prescribed dosage was 2 capsules every 24 h, with each capsule containing 9.5 mg of THC and 0.5 mg of CBD. In case patients experienced adverse effects, the dosage was halved. Out of the six patients who successfully completed the study and adhered to the six-month capsule regimen, two patients maintained a stable weight, one exhibited a weight gain of 7.7%, and three patients achieved the primary endpoint by demonstrating a weight increase exceeding 10%.In terms of overall quality of life, there were no notable differences observed before and after the administration of cannabis treatment. However, an examination of the appetite loss subscale revealed a statistically significant reduction in patient-reported complaints related to appetite loss following cannabis treatment. During the initial two weeks of cannabis treatment, the majority of patients reported an increased appetite, with approximately half noting pain reduction and improved sleep. Two patients reported enhanced mood and reduced fatigue. Nevertheless, a substantial number of patients experienced adverse effects, including tiredness, dizziness, disorientation, anxiety, hallucinations, and altered general functioning, which were associated with cannabis intake.

The use of delta-9-tetrahydrocannabinol (THC) to enhance taste and smell perception, as well as appetite, caloric intake, and overall quality of life in cancer patients, was investigated by Brisbois and colleagues [20]. Patients with advanced cancer experiencing compromised taste, smell, or both were recruited and randomly assigned to receive either 2.5 mg of dronabinol or a placebo, both administered in tablet form. After four days, the dosage was doubled, with a maximum of 20 mg per day. While the total scores obtained showed improvement compared to baseline, no significant differences were observed between the two groups, except for a few individual items. Seventy-three percent of THC-treated patients reported an increased overall appreciation of food compared to those treated with a placebo, finding it tastier than the placebo (p = 0.04). No THC-treated patients reported a decrease in appetite. In contrast, the majority of placebo-treated patients experienced a decrease in appetite (50%), or no change (20%). Caloric intake remained unchanged between the THC and placebo groups, and scores for quality of life similarly improved in both groups. Lastly, no differences in side effects emerged between the two groups.

Jatoi et al. explored the use of dronabinol, with or without megestrol acetate, for palliative care in cancer patients experiencing anorexia [18]. 469 patients (mean age 65 years) with histologic evidence of an incurable malignancy and self-reported weight loss of at least 5 pounds (2.3 kg) during the preceding 2 months and/or a physician-estimated caloric intake of less than 20 calories/kg of body weight per day were enrolled. They were randomized to oral megestrol acetate liquid suspension plus placebo capsules, oral dronabinol capsules plus liquid suspension of placebo, orboth agents. In the megestrol acetate group, 75% of patients reported an increase in their appetite at some point during the study period, whereas only 49% of patients in the dronabinol group reported a similar improvement. However, there was no statistically significant improvement observed with combination therapy when direct comparisons were made to the megestrol acetate arm. Similar results in favor of megestrol acetate alone were observed concerning weight gain: 14% of patients treated with megestrol acetate experienced a weight gain of 10% or more from their baseline, while only 5% of patients in the dronabinol group exhibited such weight gain (Fisher’s exact test, p = 0.009). Similarly, the combination of megestrol acetate and dronabinol resulted in a non-significant difference compared to the use of megestrol acetate alone. Regarding quality of life, patients treated with megestrol acetate alone appeared to have an advantage. Despite 18% of male patients reporting impotence during megestrol acetate treatment, there were no statistically different incidences of adverse events between the treatment groups.

Finally, two studies focused on the effect of nabilone on appetite and nutritional status in cancer patients [19,22]. The study by Côté and colleagues focuses on the use of nabilone vs. placebo in patients undergoing radiotherapy for histological diagnosis of squamous cell carcinoma of the oral cavity, the oropharynx, the hypopharynx, and/or the larynx [19].The administration of nabilone began the day before the first radiotherapy treatment, starting with one pill at bedtime (0.5 mg orally once daily) for the entire first week. In the second week, the dosage increased to two pills a day (0.5 mg orally twice daily). From the third week until the completion of radiotherapy treatments, the radiation oncologist adjusted the dosage, reaching a maximum of four pills a day (1 mg orally twice daily). The control group followed a corresponding procedure using a placebo. The patients enrolled were 56 (mean age 63.5 years): the use of nabilone did not improve the quality of life for these patients. In fact, no differences were recorded in pain, appetite, weight gain, nausea symptoms and sleep. However, no differences in adverse effects were observed. Turcott and colleagues analyzed the effects of nabilone administration in 33 patients with lung cancer. They were randomly assigned to receive either oral nabilone or a placebo [22]. At the 4 and 8-week evaluation, no statistically significant differences were found between the control and experimental groups concerning appetite and anthropometric variables. Patients in the experimental group, however, showed a statistically significant difference in carbohydrate consumption compared to the control group (p = 0.040) at the 8-week evaluation. The control group, on the other hand, recorded a statistically significant decrease in energy consumption. The functional, emotional, social, pain, and insomnia scales of the quality of life were better in the group treated with nabilone at the eighth week of administration. However, the control group exhibited a significant reduction in appetite loss, while the experimental group showed a difference, albeit only borderline significant (p = 0.060).

## 4 Discussion

We conducted a comprehensive systematic review of studies examining the utilization of cannabinoids in older adults to stimulate appetite. The evidence indicates a noteworthy role of these molecules in older patients with active neoplastic disease, showcasing a potential significant enhancement in the patients' quality of life through weight gain and the restoration of hunger and appetite sensations.

Studies on murine models suggest that the mechanisms underlying the orexigenic effect of cannabinoids involve the hypothalamus, CB1 receptors, and the modulation of neuropeptides and endocannabinoid systems. THC, a type of cannabinoid, has been shown to increase food intake by activating CB1 receptors [23]. These receptors are present throughout the brain, particularly in the hypothalamus, where the potent orexigenic neuropeptide Y (NPY) is also found [23]. It is likely that cannabinoids enhance the release of NPY, as they are naturally orexigenic and can potentiate neurotransmitter release [23]. Further studies have confirmed these findings, suggesting that the effect of ghrelin, another neuropeptide that stimulates appetite and regulates energy balance in the periphery, on hypothalamic AMPK, a key enzyme for energy homeostasis, on neuronal activity in the hypothalamus, and on appetite, depends on CB1 receptors [24]. These results support the existence of a signaling cascade involving ghrelin, endocannabinoids, CB1, AMPK, ultimately leading to increased appetite [24]. Based on these effects, although there is evidence of the beneficial effects of cannabinoids on appetite stimulation, it is important to further investigate how these mechanisms operate in different types of patients. This could lead to new therapies for conditions such as anorexia, involuntary weight loss, and other eating disorders. Such research is particularly relevant for subgroups of patients with chronic diseases, the elderly, or individuals with eating disorders not necessarily related to cancer-associated anorexia. Nevertheless, a notable gap in data persists regarding the application of cannabinoids in nutritional contexts, possibly exacerbated by patients frequently discontinuing studies due to adverse effects associated with both the underlying disease and its treatment. Consequently, the issue of their application remains contentious, necessitating further studies involving extensive sample sizes.

### 4.1 Use of cannabinoids in cancer patients

All patients in this review were affected by cancer. Advancing age emerges as the primary and most significant risk factor for cancer across all categories [25]. Incidence rates for cancer show a consistent rise with increasing age, peaking at a prevalence of 1,000 per 100,000 people in the age groups of 60 years and older [25]. One of the negative predictors in cancer patients is anorexia, which is associated with reduced short-term and long-term survival [26]. Anorexia is a commonly reported factor in these patients, with rates ranging from 40% to 44.5% depending on the assessment tool used [26]. Cachexia, characterized by a substantial loss of muscle and adipose tissue, is closely intertwined with anorexia. Both anorexia and weight loss play significant roles in contributing to cancer-related fatigue, functional decline, impaired survival, and treatment intolerance in cancer patients [21]. This has led to the coining of the term Cancer Anorexia-Cachexia Syndrome (CACS) to describe a profound and debilitating aspect that spans various stages of malignancy, arising from insufficient oral intake and metabolic changes [27]. CACS is the leading cause of death for 22%–30% of cancer patients, especially in older individuals, where additional age-related factors such as sarcopenia and age-related loss of muscle mass and function contribute [28]. It can also worsen chemotherapy-derived toxicity [22]. Therefore, discovering therapeutic strategies that can slow down or even halt the effects of CACS is a priority for cancer patients. Moreover, reducing the impact of side effects from therapies and consequently supporting quality of life is increasingly important, both for the benefit of patients and because certain patient groups are achieving longer survival rates today [19]. In this review, the use of cannabinoids to counteract the presence of anorexia and cachexia has been occasionally satisfactory, although the molecules employed (namely delta-9-tetrahydrocannabinol or cannabidiol or cannabis extract or nabilone) did not consistently outperform the placebo. This could be partially attributed to the low dosages of the agents used, at times necessitated by the risk of adverse events and subsequent loss of patients during follow-up in the studies [21]. It is known, albeit not entirely elucidated, that CACS has an underlying pro-inflammatory mechanism, marked by the expression of high levels of cytokines such as IL-6 [28]. Some common CACS biomarkers observed in clinical trials in humans include C-reactive protein, albumin, insulin-like growth factor-1 (IGF-1), free fatty acids, IL-6, and IL-10 [29]. However, the therapeutic or prophylactic applications of cannabinoids as anti-inflammatory agents remain controversial. A meta-analysis focusing on the role of cannabidiol, cannabigerol, and delta-9-tetrahydrocannabinol (THC) reported that only THC does not reduce pro-inflammatory cytokines, while the use of cannabidiol exerts a predominantly anti-inflammatory effect in vivo [30]. This should prompt consideration of the potential use of other molecules for the treatment of CACS. For instance, megestrol acetate has been the most studied agent for treating cancer-related anorexia, alleviating symptoms and promoting weight gain in patients with advanced-stage cancer [22]. However, a large proportion of patients continue to suffer from anorexia despite treatment with this hormone. Additionally, its long-term use is limited by the development of potentially serious side effects such as thromboembolic phenomena, edema, lower response rates to chemotherapy, and a trend for inferior survival duration [22]. Other nutritional supplements, including anamorelin, a ghrelin receptor agonist, have also been tested, yielding limited results [22]. Preserving the patient's motivation to continue ongoing therapies remains crucial, for example, by slowing down the deterioration of the individual's condition. Indeed, the longer an acceptable quality of life is maintained, the better the patient's motivation will be [31]. A better quality of life also has a positive effect on the patient's support network, reducing pressure on natural caregivers (e.g., family, friends, etc.) [32]. Additionally, it decreases the need for professional support at the beginning of therapies, optimizing available resources.

The safety and efficacy of cannabinoids, especially in the older population, remain subjects of ongoing debate. A recent systematic review has focused the available evidence on these aspects in the older adults, finding that THC and CBD, when used individually, exhibit very low rates of adverse events [33]. No general adverse events or treatment-related adverse events were reported in either treatment group, indicating good tolerability [33]. Conversely, combined THC:CBD treatment showed higher rates of serious adverse events compared to individual cannabinoids, particularly with higher doses of THC, with cases of withdrawal due to both general and treatment-related causes [33]. However, a study conducted in Switzerland on 19 patients with severe dementia revealed that THC:CBD treatment for 13 months was associated with few reported treatment-related issues and limited adverse drug reactions [34]. Overall, while exercising caution in the use of THC:CBD combined formulations, cannabinoids appear to be generally well-tolerated in the elderly, with no significant increase in the risk of serious adverse events or study withdrawal [33].

### 4.2 Lack of evidence and future perspective

An important aspect underscored by this work is indeed the lack of studies that extend beyond the neoplastic context. The older patient is not immune to involuntary weight or appetite loss, which is associated with severe physiological, psychological, and immunological consequences, irrespective of the underlying causes [33]. Not only acute illnesses but also chronic disorders, such as cognitive decline, can have significant implications for the nutritional well-being of older adults [35]. The National Institute of Neurological and Communicative Disorders and Strokes Task Force on Alzheimer's Disease (AD) includes weight loss among the clinical features consistent with a diagnosis of AD [36]. This weight loss is a multifactorial event, related not only to cognitive impairment associated with loss of appetite and reduced food intake, but also to AD-related alterations in energy consumption due to hypothalamic feeding dysregulation, olfactory changes, and psycho-behavioral disturbances [36]. Dysphagia (apraxia of swallowing), which is a common feature in the later stages of the disease, is also implicated and may worsen malnutrition [36].We hope, therefore, that our review can serve as a stimulus for conducting further clinical studies involving older patients with the primary objective of counteracting anorexia and cachexia.

## 5 Limitations

A first limitation is that the age range of patients in the included studies leans more towards the "young old" group, hence it may not be sufficiently representative of older and oldest-old individuals, where the phenomenon of anorexia of aging is more prevalent. Furthermore, while the overall quality evaluation of the studies was high, the clinical relevance remains limited due to the inclusion of small populations. This suggests the need for further investigations with larger sample sizes. Furthermore, data heterogeneity across the included studies, notably in terms of outcome measures and methodologies, precluded us from conducting a meta-analysis. Another limitation is the inclusion of only English language publications. Furthermore, the studies mainly targeted higher-income populations, which are not representative of the general population of the world, while few were carried out in low- and middle-income countries (LMIC), which may limit the applicability of the findings. In addition, some studies have possible confounders that could affect the final results, for example the high number of dropouts due to the side and adverse effects of cancer itself or cannabinoids. On the other hand, the strength of our review is the focus on older people, considering their peculiar characteristics and needs that go beyond those of the general population.

## 6 Conclusions

The evidence from our review highlights inconclusive results regarding the use of cannabinoids in older adults: despite a partial improvement in appetite and weight gain in cancer patients, results are inhomogeneous and not always reached statistically significance. Besides, there is a lack of data on the application of these treatments in non-neoplastic contexts. Future studies will be necessary to further explore the implications of cannabinoids on the quality of life, especially among older individuals.

## Author contributions

Conceptualization: Mario Virgilio Papa; Data curation: Mario Virgilio Papa, Chiara Ceolin, Adele Ravelli; Formal analysis: Mario Virgilio Papa; Methodology: Mario Virgilio Papa, Chiara Ceolin; Supervision: Marina De Rui, Maria Devita, Giuseppe Sergi, Alessandra Coin; Writing – original draft: Chiara Ceolin, Adele Ravelli; Writing – review & editing: Marina De Rui, Maria Devita, Alessandra Coin, Chiara Ceolin, Adele Ravelli

## Ethical approval

Ethical approval was not required for this secondary research.

## Funding

This research did not receive any specific grant from funding agencies in the public, commercial, or not-for-profit sectors.

## Competing interests

All authors state that they have no competing interests.
